# Central Upwind Scheme for a Compressible Two-Phase Flow Model

**DOI:** 10.1371/journal.pone.0126273

**Published:** 2015-06-03

**Authors:** Munshoor Ahmed, M. Rehan Saleem, Saqib Zia, Shamsul Qamar

**Affiliations:** Department of Mathematics, COMSATS Institute of Information Technology, Islamabad, Pakistan; China University of Mining and Technology, CHINA

## Abstract

In this article, a compressible two-phase reduced five-equation flow model is numerically investigated. The model is non-conservative and the governing equations consist of two equations describing the conservation of mass, one for overall momentum and one for total energy. The fifth equation is the energy equation for one of the two phases and it includes source term on the right-hand side which represents the energy exchange between two fluids in the form of mechanical and thermodynamical work. For the numerical approximation of the model a high resolution central upwind scheme is implemented. This is a non-oscillatory upwind biased finite volume scheme which does not require a Riemann solver at each time step. Few numerical case studies of two-phase flows are presented. For validation and comparison, the same model is also solved by using kinetic flux-vector splitting (KFVS) and staggered central schemes. It was found that central upwind scheme produces comparable results to the KFVS scheme.

## Introduction

Multiphase flows are commonly observed in nature and science, from stand storms, to volcanic clouds, blood flow in vessels and motion of rain droplets. There are also numerous examples where multiphase flow occurs in industrial applications, for example energy conversion, paper manufacturing, food manufacturing as well as in chemical and process engineering. Due to their wide range applications, suitable models are required for the accurate prediction of the physical behavior of such flows. However, modeling and simulation of flows is a complex and challenging research area of the computational fluid dynamics (CFD).

Multiphase flow problems involve the flow of two or more fluids separated by sharp interfaces. The coupling of interfaces with the flow model is a challenging part in the simulation of such flows, as coupling miss-match may introduce large errors in the numerical simulations. It is important to mention that this work is only concerned with two-phase flow problem.

Several two-phase flow models exist in the literature for describing the behavior of physical mixtures. These models use separate pressures, velocities and densities for each fluid. Moreover, a convection equation for the interface motion is coupled with the conservation laws of flow models. In the literature such models are known as seven-equation models. One of such models for solid-gas two-phase flows was initially introduced by Baer and Nunziato [1] and was further investigated by Abgrall and Saurel [2, 3], among others. The seven-equation model is considered as the best and established two-phase flow model. However, it inherit a number of numerical complexities. To resolve such difficulties researchers have proposed reduced models containing three to six equations [4–6].

The Kapila’s five-equation model [4] deduced from Baer and Nunziato seven-equation model [1] is a well known reduced model and has been successfully implemented to study interfacing compressible fluids, barotropic and non-barotropic cavitating flows. The model contains four conservative equations, two for mass conservation, one for total momentum and one for total energy conservation. The fifth equation is a non-zero convection equation for the volume fraction of one of two phases.

Although, this five-equation model is simple, but, it involves a number of serious difficulties. For example, the model is non-conservative and hence it is difficult to obtain a numerical solution which converges to the physical solution. In the presence of shocks, the volume fraction may become negative. Another issue is related to non-conservative behavior of the mixture sound speed [7].

To overcome the associated difficulties of Kapila’s five-equation model, Kreeft and Koren [5] introduced a new formulation of the Kapila’s model. The new model [5] is also non-conservative containing two equations for the conservation of mass, one each for mixture momentum and total energy respectively. The fifth equation is the energy equation for one of the two phases and it includes source term on the right hand side which represents the energy exchange between two fluids in the form of mechanical and thermodynamical work. In the current model, the first four equations are conservative and the non-conservativity in the models is due to the energy exchange term in the fifth equation. Consequently, the implementation of finite volume type schemes are relatively convenient to such models.

Very recently, diffuse interface method, finite volume WENO scheme and discontinuous Galerkin method have been used to solve the multiphase flow models [8–10]. However, in this work, the central upwind scheme [11] is proposed to solve the same five-equation model [5]. The proposed scheme uses information of local propagation speeds and estimates the solution in terms of cell averages. Further, the scheme has an upwind nature, because it takes care of flow directions by means of one-sided local speeds. Moreover, this scheme can be extended to incompressible flow problems e.g. it can be extended to solve incompressible two-phase shallow flow model [12]. The suggested scheme is applied to both one and two-dimensional flow models. For validation, the results of central upwind scheme are compared with those obtained from the KFVS [13–16] and the non-oscillatory staggered central scheme [17, 18]. The numerical results of the schemes are analyzed qualitatively and quantitatively. It was found that the proposed central upwind scheme has comparable results to the KFVS scheme and are more accurate than the staggered central scheme.

## One-dimensional two-fluid flow model

In this section, the one-dimensional two-fluid flow model [5] is presented. The selected model is the reformulation of original five-equation model of Kapila et al. [4]. Here, it is assumed that both fluids are mass conservative and have same velocity and pressure on both sides the interface. Moreover, heat conduction and viscosity are not considered. In this model, first four equations describe the conservative quantities: two for mass, one for over all momentum and one for total energy. The fifth equation is the energy equation and it includes source term on the right hand side which gives the energy exchange between two fluids in the form of mechanical and thermodynamical work. The state vector **q** of primitive variables has the form **q** = (*ρ*, **u**, *p*, *α*)^*T*^. Here, the bulk mixture density is denoted by *ρ*, **u** = (*u*, 0, 0) are the bulk velocities along each characteristic direction, *p* denotes the bulk pressure and *α* represents the volume fraction of fluid 1. This means that a part *α* of a small volume *dV* is filled with fluid 1 and a part (1—*α*) with fluid 2.

For bulk quantities, such as mixture density *ρ* and mixture total energy *E*, we assume that *α* is a volume fraction of fluid 1 and (1—*α*) of fluid 2. Using these conventions, we can define

$$\begin{array}{c} \rho =\alpha \rho_{1}+\left(1-\alpha \right)\rho_{2}\;,\;\rho E=\alpha \rho_{1}E_{1}+\left(1-\alpha \right)\rho_{2}E_{2} \end{array}$$ (1)

and the total energies of each fluid as

$$\begin{array}{c} E_{1}=e_{1}+\frac{1}{2}u^{2}\;,\;E_{2}=e_{2}+\frac{1}{2}u^{2}, \end{array}$$ (2)

where *e*_1_ and *e*_2_ denote the internal energies of fluid 1 and fluid 2, respectively. The internal energies *e*_1_ and *e*_2_ are given in terms of their respective densities and pressure through equations of state

$$\begin{array}{c} e_{1}=e_{1}\;(\rho_{1},p),\;e_{2}=e_{2}\;(\rho_{2},p). \end{array}$$ (3)

In one space dimensions, the two-fluid flow model can be written as [5]

$$\begin{array}{c} w_{t}+{f(w)}_{x}=s\;, \end{array}$$ (4a)

where

$$\begin{array}{c} w={\left(\rho ,\rho u,\rho E,\rho_{1}\alpha ,\rho_{1}E_{1}\alpha \right)}^{T}, \end{array}$$ (4b)

$$\begin{array}{c} f\left(w\right)={\left(\rho u,\rho u^{2}+p,\rho uE+pu,\rho_{1}u\alpha ,\rho_{1}E_{1}u\alpha +pu\alpha \right)}^{T}, \end{array}$$ (4c)

$$\begin{array}{c} \;s\left(w\right)={\left(0,0,0,0,0,s_{5}\right)}^{T}. \end{array}$$ (4d)

Here, **w** represents the vector of conservative variables, **f** is the vectors of fluxes, **s** is a vector of source terms with only last non-zero term. The term *s*_5_ represents the total rate of energy exchange per unit volume between fluid 1 and fluid 2 and is equal to the sum of rates of mechanical ${s_{5}}^{M}$ and thermodynamic ${s_{5}}^{T}$ works [5], i.e. $s_{5}={s_{5}}^{M}+{s_{5}}^{T}$, with

$$\begin{array}{c} {s_{5}}^{M}=u{\left(p\alpha \right)}_{x}-\beta up_{x}, \end{array}$$ (5)

$$\begin{array}{c} {s_{5}}^{T}=p\alpha \left(1-\alpha \right)\frac{\tau_{2}-\tau_{1}}{\tau }u_{x}\;. \end{array}$$ (6)

The term $\beta =\frac{\rho_{1}\alpha }{\rho }$ represents the mass fraction of fluid 1, while the relations $\tau_{1}=\frac{1}{\rho_{1}c_{1}^{2}}$ and $\tau_{2}=\frac{1}{\rho_{2}c_{2}^{2}}$ denote the isentropic compressibilities of both fluids. Here, *c*_1_ and *c*_2_ represent the sound speeds of fluid 1 and fluid 2. The bulk isentropic compressibility is defined as

$$\begin{array}{c} \tau =\alpha \tau_{1}+\left(1-\alpha \right)\tau_{2}\;. \end{array}$$ (7)

Assume that the equations of state in Eq (3) are the stiffened equations of state [19]

$$\begin{array}{c} \rho_{i}e_{i}=\frac{p+\pi_{i}\gamma_{i}}{\gamma_{i}-1},\;i=1,2\;, \end{array}$$ (8)

where *γ*_*i*_, *π*_*i*_ are the material specific quantities. Therefore, the sound speeds in each fluid are given as

$$\begin{array}{c} c_{i}=\sqrt{\frac{\left(p+\pi_{i}\right)\gamma_{i}}{\rho_{i}}},\;i=1,2\;. \end{array}$$ (9)

The expressions for the sound speeds are normally obtained from the second law of thermodynamics. The total energies of fluids 1 and 2 can be given as

$$\begin{array}{c} \rho_{1}E_{1}\alpha =\frac{p+\pi_{1}\gamma_{1}}{\gamma_{1}-1}\alpha +\frac{1}{2}\rho_{1}\alpha u^{2}\;, \end{array}$$ (10)

$$\begin{array}{c} \rho_{2}E_{2}\left(1-\alpha \right)=\frac{p+\pi_{2}\gamma_{2}}{\gamma_{2}-1}\left(1-\alpha \right)+\frac{1}{2}\left(\rho -\rho_{1}\alpha \right)u^{2}\;. \end{array}$$ (11)

Using Eqs (4), (10) and (11), we obtain the primitive variables as

$$\begin{array}{c} \rho =w_{1},\;u=\frac{w_{2}}{w_{1}}\;, \end{array}$$ (12)

$$\begin{array}{c} \alpha =\{\begin{array}{c} \frac{\beta_{1}}{\beta_{1}+\beta_{2}}\;,\;\text{if}\;\;\;\pi_{1}=0=\pi_{2}\;,\\ \frac{\pi_{2}\gamma_{2}-\pi_{1}\gamma_{1}-\beta_{1}-\beta_{2}\pm \sqrt{{\left(\pi_{2}\gamma_{2}-\pi_{1}\gamma_{1}-\beta_{1}-\beta_{2}\right)}^{2}+4\beta_{1}\left(\pi_{2}\gamma_{2}-\pi_{1}\gamma_{1}\right)}}{2(\pi_{2}\gamma_{2}-\pi_{1}\gamma_{1})}\;,\;\text{otherwise}\;, \end{array} \end{array}$$ (13)

$$\begin{array}{c} p=\beta_{1}+\beta_{2}-\alpha \pi_{1}\gamma_{1}-\left(1-\alpha \right)\pi_{2}\gamma_{2}\;, \end{array}$$ (14)

where

$$\begin{array}{c} \beta_{1}=\left(\gamma_{1}-1\right)\;(w_{5}-\frac{w_{4}\left(w_{2}^{2}\right)}{2w_{1}^{2}})\;, \end{array}$$ (15)

$$\begin{array}{c} \beta_{2}=\left(\gamma_{2}-1\right)\;(w_{3}-w_{5}-\frac{\left(w_{1}-w_{4}\right)\left(w_{2}^{2}\right)}{2w_{1}^{2}})\;. \end{array}$$ (16)

Here, *w*_*i*_; *i* = 1,…,5, represent the components of **w**, the vector of conserved variables. Moreover, in Eqs (12)–(14) the primitive variables are expressed in terms of conserved variables. While in Eq (13) the positive sign is chosen for (*π*_2_ *γ*_2_−*π*_1_ *γ*_1_) > 0 and negative otherwise. Because of Eq (9)

$$\begin{array}{c} \tau_{1}=\frac{1}{\rho_{1}c_{1}^{2}}=\frac{1}{\left(p+\pi_{1}\right)\gamma_{1}}\;,\;\tau_{2}=\frac{1}{\rho_{2}c_{2}^{2}}=\frac{1}{\left(p+\pi_{2}\right)\gamma_{2}}\;. \end{array}$$ (17)

## One dimensional Central upwind scheme

In this section, the central upwind scheme of Kurganov and Tadmor [11] is derived for the one-dimensional five-equation two-fluid flow model Eq (1). Let *N* represents the total number of discretization points and ${\left(x_{i-\frac{1}{2}}\right)}_{i}\in \{1,\cdots ,N+1\}$ denotes the divisions of the given domain [0, *x*_*max*_]. A uniform width Δ*x* for each cell is considered, while, *x*_*i*_ represents the cell centers and $x_{i+\frac{1}{2}}$ refer to the cell boundaries.

Further, we take

$$\begin{array}{c} x_{\frac{1}{2}}=0,\;\;\;\;x_{N+\frac{1}{2}}=x_{max},\;\;\;\;x_{i+\frac{1}{2}}=i\cdot \Delta x. \end{array}$$ (18)

Moreover,

$$\begin{array}{c} x_{i}=\frac{x_{i-\frac{1}{2}}+x_{i+\frac{1}{2}}}{2}\;\;\text{and}\;\;\Delta x=x_{i+\frac{1}{2}}-x_{i-\frac{1}{2}}=\frac{x_{max}}{N+1}. \end{array}$$ (19)

Let $\Omega_{i}:=\left[x_{i-\frac{1}{2}},x_{i+\frac{1}{2}}\right]$ for *i* ≥ 1. The cell average values of conservative variables **w**_*i*_ are defined as

$$\begin{array}{c} w_{i}:=w_{i}\left(t\right)=\frac{1}{\Delta x}\int_{\Omega_{i}}w\left(t,x\right)dx\;. \end{array}$$ (20)

Integration of Eq (4) over the interval Ω_*i*_ gives

$$\begin{array}{c} \frac{dw_{i}\left(t\right)}{dt}=-\frac{H_{i+\frac{1}{2}}\;\left(t\right)-H_{i-\frac{1}{2}}\;\left(t\right)}{\Delta x}+\frac{s_{i}\left(t\right)}{\Delta x}\;, \end{array}$$ (21)

where $H_{i+\frac{1}{2}}(t)$ is the numerical flux defined by

$$\begin{array}{c} H_{i\pm \frac{1}{2}}\;\left(t\right)=\frac{f\left(w_{i\pm \frac{1}{2}}^{+}\right)\left(t\right)+f\left(w_{i\pm \frac{1}{2}}^{-}\right)\left(t\right)}{2}-\frac{a_{i\pm \frac{1}{2}}}{2}\left[w_{i\pm \frac{1}{2}}^{+}\;\left(t\right)-w_{i\pm \frac{1}{2}}^{-}\;\left(t\right)\right]\;. \end{array}$$ (22)

The first four components of the source vector **s**_*i*_ in Eq (21) are zero and the fifth non-zero component is given as

$$\begin{array}{ccc} s_{5i}\left(t\right) & = & \frac{\left(u_{i+\frac{1}{2}}^{-}+u_{i-\frac{1}{2}}^{+}\right)}{2}\cdot \left({\left(p\alpha \right)}_{i+\frac{1}{2}}^{-}-{\left(p\alpha \right)}_{i-\frac{1}{2}}^{+}\right)\\ & & +\;\frac{\left(\beta_{i+\frac{1}{2}}^{-}+\beta_{i-\frac{1}{2}}^{+}\right)}{2}\frac{\left(u_{i+\frac{1}{2}}^{-}+u_{i-\frac{1}{2}}^{+}\right)}{2}\cdot \left({\left(p\right)}_{i+\frac{1}{2}}^{-}-{\left(p\right)}_{i-\frac{1}{2}}^{+}\right)\\ & & +\;\frac{\left(\eta_{i+\frac{1}{2}}^{-}+\eta_{i-\frac{1}{2}}^{+}\right)}{2}\cdot \left(u_{i+\frac{1}{2}}^{-}-u_{i-\frac{1}{2}}^{+}\right)\;, \end{array}$$ (23)

where $\eta =p\alpha (1-\alpha )\frac{\tau_{2}-\tau_{1}}{\tau }u_{x}$.

The intermediate values ${\text{w}}_{i+\frac{1}{2}}^{-}$ and ${\text{w}}_{i+\frac{1}{2}}^{+}$ are given as

$$\begin{array}{c} w_{i+\frac{1}{2}}^{-}=w_{i}-\frac{1}{2}w_{i}^{x}\;,\;\;w_{i+\frac{1}{2}}^{+}=w_{i}+\frac{1}{2}w_{i}^{x}\;. \end{array}$$ (24)

The numerical derivatives ${\text{w}}_{i}^{x}$ are approximated through a nonlinear limiter which guarantees the positivity of the reconstruction procedure Eq (24).

$$\begin{array}{c} w_{i}^{x}=MM\{\theta \Delta w_{i+\frac{1}{2}},\frac{\theta }{2}\;(\Delta w_{i+\frac{1}{2}}+\Delta w_{i-\frac{1}{2}}),\theta \Delta w_{i-\frac{1}{2}}\}\;. \end{array}$$ (25)

Here, *MM* denotes the min-mod non-linear limiter

$$\begin{array}{c} MM\left\{x_{1},x_{2},...\right\}=\{\begin{array}{c} {\text{min}}_{i}\left\{x_{i}\right\}\;\text{if}\;\;x_{i}>0\;\forall i\;,\\ {\text{max}}_{i}\left\{x_{i}\right\}\;\text{if}\;\;x_{i}<0\;\forall i\;,\\ 0\;\;\text{otherwise}\;. \end{array} \end{array}$$ (26)

Moreover, $a_{i+\frac{1}{2}}(t)$ represents the maximal local which in the generic case could be

$$\begin{array}{c} a_{i+\frac{1}{2}}\;\left(t\right):=\max\{\rho \;(\frac{\partial f}{\partial w}\left(w_{i+\frac{1}{2}}^{-}\right)\left(t\right)),\rho \;(\frac{\partial f}{\partial w}\left(w_{i+\frac{1}{2}}^{+}\right)\left(t\right))\}\;. \end{array}$$ (27)

To achieve the second order accuracy in time, a second order TVD RK-method is applied to the Eq (21). For simplicity if the right hand side of the Eq (21) is taken as *L*(**w**), then two stage TVD RK-method to update **w** is given as under

$$\begin{array}{c} w^{\left(1\right)}=w^{n}+\Delta tL\left(w^{n}\right)\;, \end{array}$$ (28a)

$$\begin{array}{c} w^{n+1}=\frac{1}{2}(w^{n}+w^{\left(1\right)}+\Delta t\;L\left(w^{\left(1\right)}\right))\;, \end{array}$$ (28b)

where **w** ^*n*^ is a solution at previous time step and **w** ^*n*+1^ is updated solution at next time step. Moreover, Δ*t* represents the time step.

## Two-dimensional two-fluid flow model

In two-dimensional space, the two-fluid flow model can be written as [5]

$$\begin{array}{c} w_{t}+f\;{\left(w\right)}_{x}+g{\left(w\right)}_{y}=s\;, \end{array}$$ (29a)

where

$$\begin{array}{c} w={\left(\rho ,\rho u,\rho v,\rho E,\rho_{1}\alpha ,\rho_{1}E_{1}\alpha \right)}^{T}, \end{array}$$ (29b)

$$\begin{array}{c} f\;\left(w\right)=(\rho u,\rho u^{2}+p,\rho uv,\rho uE+pu,\rho_{1}u\alpha ,\rho_{1}E_{1}u\alpha +pu\alpha ),\; \end{array}$$ (29c)

$$\begin{array}{c} g\left(w\right)=(\rho v,\rho vu,\rho v^{2}+p,\rho vE+pv,\rho_{1}u\alpha ,\rho_{1}E_{1}v\alpha +pv\alpha )\;, \end{array}$$ (29d)

$$\begin{array}{c} \;s\left(w\right)=(0,0,0,0,0,s_{6}). \end{array}$$ (29e)

Here, **w** represents the vector of conservative variables, **f**, **g** are vectors of fluxes in *x* and *y* directions, **s** is a vector of source terms with only last non-zero term. The term *s*_6_ represents the total rate of energy exchange per unit volume between fluid 1 and fluid 2 and is equal to the sum of rates of mechanical ${s_{6}}^{M}$ and thermodynamic ${s_{6}}^{T}$ work [5], i.e. $s_{6}={s_{6}}^{M}+{s_{6}}^{T}$, with

$$\begin{array}{c} {s_{6}}^{M}=u{\left(p\alpha \right)}_{x}+v{\left(p\alpha \right)}_{y}-\beta up_{x}-\beta vp_{y}, \end{array}$$ (30)

$$\begin{array}{c} {s_{6}}^{T}=p\alpha \left(1-\alpha \right)\frac{\tau_{2}-\tau_{1}}{\tau }\left(u_{x}+v_{y}\right)\;. \end{array}$$ (31)

Let $|\text{u}|:=\sqrt{u^{2}+v^{2}}$, the total energies of fluids 1 and 2 are given as

$$\begin{array}{c} \rho_{1}E_{1}\alpha =\frac{p+\Pi_{1}\gamma_{1}}{\gamma_{1}-1}\alpha +\frac{1}{2}\rho_{1}\alpha {\left|u\right|}^{2}\;, \end{array}$$ (32)

$$\begin{array}{c} \rho_{2}E_{2}\left(1-\alpha \right)=\frac{p+\Pi_{2}\gamma_{2}}{\gamma_{2}-1}\left(1-\alpha \right)+\frac{1}{2}\left(\rho -\rho_{1}\alpha \right){\left|u\right|}^{2}\;. \end{array}$$ (33)

Since the energy equation is directional independent, therefore for one- and two-dimensional problems the procedure of calculating primitive variables are the same. In two-dimensional space, the primitive variables can be retrieved in the following manner. Using Eqs (29), (32) and (33), we obtain

$$\begin{array}{c} \rho =w_{1},\;u=\frac{w_{2}}{w_{1}}\;,\;v=\frac{w_{3}}{w_{1}}\;, \end{array}$$ (34)

$$\begin{array}{c} \alpha =\{\begin{array}{c} \frac{\beta_{1}}{\beta_{1}+\beta_{2}}\;,\;\text{if}\;\;\;\Pi_{1}=0=\Pi_{2}\;,\\ \frac{\Pi_{2}\gamma_{2}-\Pi_{1}\gamma_{1}-\beta_{1}-\beta_{2}\pm \sqrt{{\left(\Pi_{2}\gamma_{2}-\Pi_{1}\gamma_{1}-\beta_{1}-\beta_{2}\right)}^{2}+4\beta_{1}\left(\Pi_{2}\gamma_{2}-\Pi_{1}\gamma_{1}\right)}}{2(\Pi_{2}\gamma_{2}-\Pi_{1}\gamma_{1})}\;,\;\text{otherwise}\;, \end{array} \end{array}$$ (35)

$$\begin{array}{c} p=\beta_{1}+\beta_{2}-\alpha \Pi_{1}\gamma_{1}-\left(1-\alpha \right)\Pi_{2}\gamma_{2}\;, \end{array}$$ (36)

where

$$\begin{array}{c} \beta_{1}=\left(\gamma_{1}-1\right)\;(w_{6}-\frac{w_{5}\;\left(w_{2}^{2}+w_{3}^{2}\right)}{2w_{1}^{2}})\;, \end{array}$$ (37)

$$\begin{array}{c} \beta_{2}=\left(\gamma_{2}-1\right)\;(w_{4}-w_{6}-\frac{\left(w_{1}-w_{5}\right)\left(w_{2}^{2}+w_{3}^{2}\right)}{2w_{1}^{2}})\;. \end{array}$$ (38)

Similarly, as in case of one-dimensional model, *w*_*i*_; *i* = 1,…,6, represent the components of **w**, the vector of conserved variables. In Eq (35) the positive sign is chosen for (Π_2_ *γ*_2_−Π_1_ *γ*_1_) > 0 and negative otherwise.

## Two dimensional Central upwind scheme

In this section, the central upwind scheme [11] is extended to two-dimensional five-equation two-phase flow model Eq (1). To implement the scheme, first we need to discretize the computational domain.

Let *N*_*x*_ and *N*_*y*_ be the large integers in *x* and *y*-directions, respectively. We consider a cartesian grid with rectangular domain [*x*_0_, *x*_*max*_] × [*y*_0_, *y*_*max*_] and it is covered by the cells $C_{ij}\equiv \left[x_{i-\frac{1}{2}},x_{i+\frac{1}{2}}\right]\times \left[y_{i-\frac{1}{2}},y_{i+\frac{1}{2}}\right]$ where 1 ≤ *i* ≤ *N*_*x*_ and 1 ≤ *j* ≤ *N*_*y*_. Here, the representative coordinates in a cell *C*_*ij*_ are denoted by (*x*_*i*_, *y*_*j*_).

Further, we take

$$\begin{array}{c} \left(x_{\frac{1}{2}},y_{\frac{1}{2}}\right)=\left(0,0\right),\;\;\;\;x_{i}=\frac{x_{i-\frac{1}{2}}+x_{i+\frac{1}{2}}}{2},\;\;\;\;y_{j}=\frac{x_{j-\frac{1}{2}}+x_{j+\frac{1}{2}}}{2}, \end{array}$$ (39)

and

$$\begin{array}{c} \Delta x_{i}=\frac{x_{i+\frac{1}{2}}+x_{i-\frac{1}{2}}}{2},\;\;\;\;\Delta y_{j}=y_{j+\frac{1}{2}}-y_{j-\frac{1}{2}}. \end{array}$$ (40)

The cell average values conservative variable **w**_*i*, *j*_ at any time *t* are given as

$$\begin{array}{c} w_{i,j}:=w_{i,j}\left(t\right)=\frac{1}{\Delta x_{i}\Delta y_{j}}\int_{C_{ij}}w\left(t,x,y\right)dydx\;. \end{array}$$ (41)

The following linear piecewise interpolant is constructed as under

$$\begin{array}{c} w\left(x,y,t\right)=\sum_{i,j}\;[w_{i,j}+w_{i,j}^{x}\left(x-x_{i}\right)+w_{i,j}^{y}\left(y-y_{j}\right)]\;\chi_{i,j}. \end{array}$$ (42)

Here, *χ*_*i*, *j*_ is the characteristics function corresponding to the cell $\left(x_{i-\frac{1}{2}},x_{i+\frac{1}{2}}\right)\times \left(y_{i-\frac{1}{2}},y_{i+\frac{1}{2}}\right)$, ${\left(w\right)}_{i,j}^{x}$ and ${\left(w\right)}_{i,j}^{y}$ are the approximations of *x* and *y*-derivatives of **w** at cell centers (*x*_*i*_, *y*_*j*_). In two-dimensional case to compute the derivative a generalized *MM* limiter is used

$$\begin{array}{c} {\left(w^{x}\right)}_{i,j}^{n}=MM\;(\theta \frac{w_{i+1,j}-w_{i,j}}{\Delta x}\;,\frac{w_{i+1,j}-w_{i-1,j}}{2\Delta x}\;,\theta \frac{w_{i,j}-w_{i-1,j}}{\Delta x})\;, \end{array}$$ (43)

$$\begin{array}{c} {\left(w^{y}\right)}_{i,j}^{n}=MM\;(\theta \frac{w_{i,j+1}-w_{i,j}}{\Delta y}\;,\frac{w_{i,j+1}-w_{i,j-1}}{2\Delta y}\;,\theta \frac{w_{i,j}-w_{i,j-1}}{\Delta y}). \end{array}$$ (44)

Here, 1 ≤ *θ* ≤ 2 and *MM* is defined in Eq (26). Integration of Eq (29) over the cell *C*_*ij*_ gives the following two-dimensional extended scheme

$$\begin{array}{c} \frac{dw_{i,j}\left(t\right)}{dt}=-\frac{H_{i+\frac{1}{2},j}^{x}\left(t\right)-H_{i-\frac{1}{2},j}^{x}\left(t\right)}{\Delta x}-\frac{H_{i,j+\frac{1}{2}}^{y}\left(t\right)-H_{i,j-\frac{1}{2}}^{y}\left(t\right)}{\Delta y} \end{array}$$ (45)

$$\begin{array}{c} +\frac{s_{i,j}^{x}\left(t\right)}{\Delta x}+\frac{s_{i,j}^{y}\left(t\right)}{\Delta y}\;. \end{array}$$ (46)

Here,

$$\begin{array}{c} H_{i+\frac{1}{2},j}^{x}\left(t\right)=\frac{f\;\left(w_{i+\frac{1}{2},j}^{+}\right)\left(t\right)+f\;\left(w_{i+\frac{1}{2},j}^{-}\right)\left(t\right)}{2}-\frac{a_{i+\frac{1}{2},j}^{x}}{2}\left[w_{i+\frac{1}{2},j}^{+}\left(t\right)-w_{i+\frac{1}{2},j}^{-}\left(t\right)\right]\;, \end{array}$$ (47)

$$\begin{array}{c} H_{i,j+\frac{1}{2}}^{y}\left(t\right)=\frac{g\;\left(w_{i,j+\frac{1}{2}}^{+}\right)\left(t\right)+g\;\left(w_{i,j+\frac{1}{2}}^{-}\right)\left(t\right)}{2}-\frac{a_{i,j+\frac{1}{2}}^{y}}{2}\left[w_{i,j+\frac{1}{2}}^{+}\left(t\right)-w_{i,j+\frac{1}{2}}^{-}\left(t\right)\right]\;, \end{array}$$ (48)

and for the non-zero component of the source term is

$$\begin{array}{ccc} s_{6i,j}^{x}\left(t\right) & = & \frac{\left(u_{i+\frac{1}{2},j}^{-}+u_{i-\frac{1}{2},j}^{+}\right)}{2}\cdot \left({\left(p\alpha \right)}_{i+\frac{1}{2},j}^{-}-{\left(p\alpha \right)}_{i-\frac{1}{2},j}^{+}\right)\\ & & +\;\frac{\left(\beta_{i+\frac{1}{2},j}^{-}+\beta_{i-\frac{1}{2},j}^{+}\right)}{2}\frac{\left(u_{i+\frac{1}{2},j}^{-}+u_{i-\frac{1}{2},j}^{+}\right)}{2}\cdot \left({\left(p\right)}_{i+\frac{1}{2},j}^{-}-{\left(p\right)}_{i-\frac{1}{2},j}^{+}\right)\\ & & +\;\frac{\left(\eta_{i+\frac{1}{2},j}^{-}+\eta_{i-\frac{1}{2},j}^{+}\right)}{2}\cdot \left(u_{i+\frac{1}{2},j}^{-}-u_{i-\frac{1}{2},j}^{+}\right). \end{array}$$ (49)

$$\begin{array}{ccc} s_{6i,j}^{y}\left(t\right) & = & \frac{\left(u_{i,j+\frac{1}{2}}^{-}+u_{i,j-\frac{1}{2}}^{+}\right)}{2}\cdot \left({\left(p\alpha \right)}_{i,j+\frac{1}{2}}^{-}-{\left(p\alpha \right)}_{i,j-\frac{1}{2}}^{+}\right)\\ & & +\;\frac{\left(\beta_{i,j+\frac{1}{2}}^{-}+\beta_{i,j-\frac{1}{2}}^{+}\right)}{2}\frac{\left(u_{i,j+\frac{1}{2}}^{-}+u_{i,j-\frac{1}{2}}^{+}\right)}{2}\cdot \left({\left(p\right)}_{i,j+\frac{1}{2}}^{-}-{\left(p\right)}_{i,j-\frac{1}{2}}^{+}\right)\\ & & +\;\frac{\left(\eta_{i,j+\frac{1}{2}}^{-}+\eta_{i,j-\frac{1}{2}}^{+}\right)}{2}\cdot \left(u_{i,j+\frac{1}{2}}^{-}-u_{i,j-\frac{1}{2}}^{+}\right). \end{array}$$ (50)

The intermediate values in the present case are given as

$$\begin{array}{ccc} w_{i+\frac{1}{2},j}^{-} & = & w_{i,j}-\frac{\Delta x}{2}w_{i,j}^{x}\;,\;\;w_{i+\frac{1}{2},j}^{+}=w_{i,j}+\frac{\Delta x}{2}w_{i,j}^{x}\;,\\ w_{i,j+\frac{1}{2}}^{-} & = & w_{i,j}-\frac{\Delta y}{2}w_{i,j}^{y}\;,\;\;w_{i,j+\frac{1}{2}}^{+}=w_{i,j}+\frac{\Delta y}{2}w_{i,j}^{y}\;. \end{array}$$ (51)

Further, $a_{i+\frac{1}{2},j}^{x}(t)$ and $a_{i,j+\frac{1}{2}}^{y}(t)$ are the maximal local which are given as

$$\begin{array}{c} a_{i+\frac{1}{2},j}^{x}\left(t\right)=\max_{\pm }\rho \;(\frac{\partial f}{\partial w}\left(w_{i+\frac{1}{2},j}^{\pm }\right))\;,\;a_{i,j+\frac{1}{2}}^{y}\left(t\right)=\max_{\pm }\rho \;(\frac{\partial g}{\partial w}\left(w_{i,j+\frac{1}{2}}^{\pm }\right))\;. \end{array}$$ (52)

For details and complete derivation of the scheme, see [11].

## Numerical test problems

This section presents some numerical test problems (both one and two-dimensional) to check the capability of central upwind and KFVS schemes to compressible two-phase reduced five-equation flow model. The results are compared with those obtained from central scheme [18].

### One-dimensional test problems

In this section six one-dimensional test problems are considered to verify the efficiency and accuracy of the proposed schemes.

#### Sod’s problem

The Sod’s problem [6] is the well known test problem in the single phase gas dynamics. In this problem, gases are separated by a very thin membrane placed at *x* = 0.5 and are initially at rest. The left side gas has high density and pressure as compared to right side gas. After removing the membrane, the gases evolution in time take place. The initial data for the problem are given as

$$\begin{array}{c} (\rho ,u,p,\alpha )=(10,0,10,1)\;,\;\text{if}\;\;\;x\leq 0.5\;, \end{array}$$ (53)

$$\begin{array}{c} (\rho ,u,p,\alpha )=(0.125,0,0.1,0)\;,\;\text{if}\;\;\;x>0.5\;. \end{array}$$ (54)

**Fig 1:**
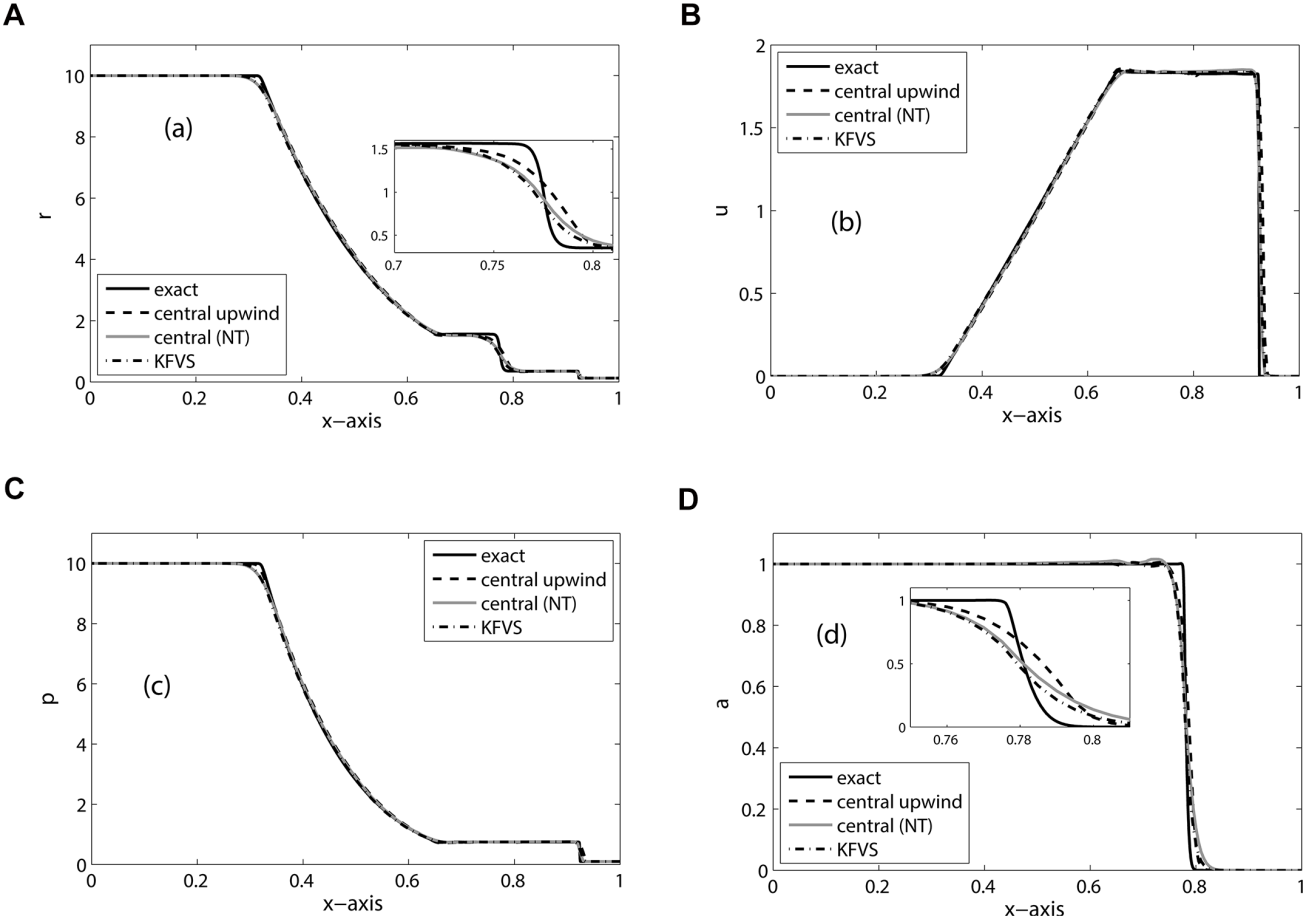
Results of Sod’s problem on 400 mesh cells at *t* = 0.015.

**Fig 2:**
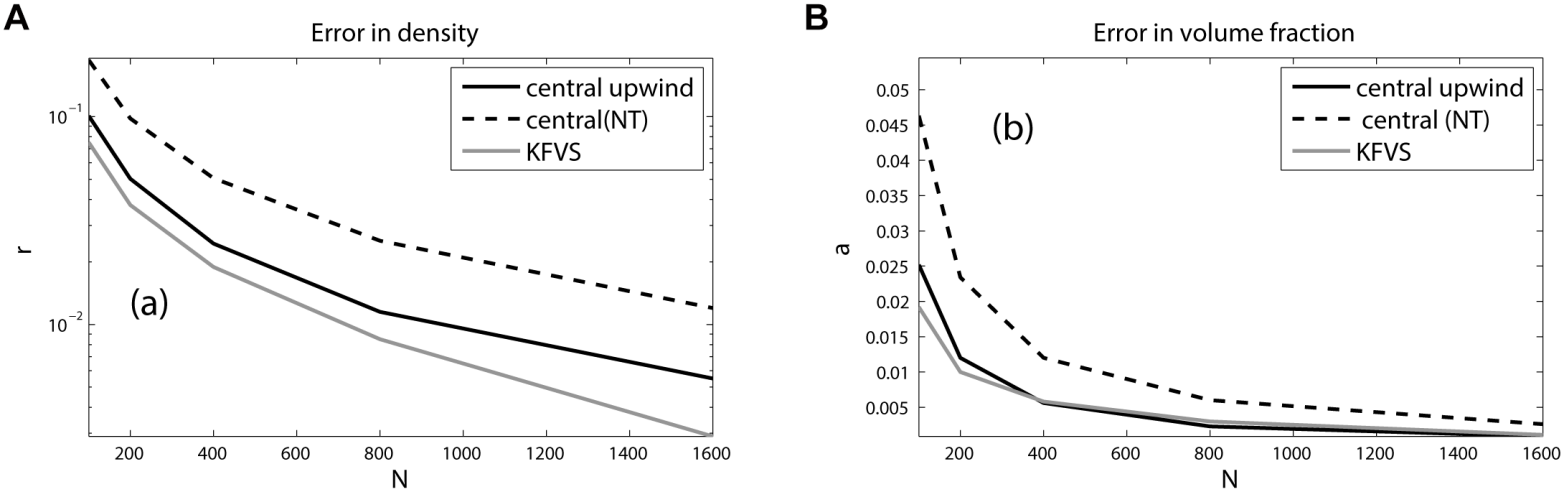
Errors in density and volume fraction.

The ratio of specific heats for the left and right side gases are taken as *γ*_*L*_ = 1.4 and *γ*_*R*_ = 1.6, respectively. The Fig 1 shows the numerical results on 400 mesh cells at *t* = 0.015. We can observe a left-going rarefaction wave, right-going shock wave and the right-moving two-fluid interface in the solution. In Fig 1, the solutions of central upwind and KFVS schemes are compared. The reference solution is obtained from the same central upwind scheme at 2000 grid points. Both schemes give correct location of the discontinuities and have comparable accuracy. Moreover, no pressure oscillations are observed in the solution. Further, Fig 2 shows the graphical representation of the errors in density and volume fraction. The results show that KFVS scheme produces less error in density solutions compared to the central upwind and central schemes. Moreover, in volume fraction solution the central upwind scheme produces less error compared to the other two schemes.

#### Two-fluid mixture problem

The initial data are given as

$$\begin{array}{c} (\rho ,u,p,\alpha )=(2.0,0,1000,1)\;,\;\text{if}\;\;\;x\leq 0.5, \end{array}$$ (55)

$$\begin{array}{c} (\rho ,u,p,\alpha )=(1,0,0.01,0)\;,\;\text{if}\;\;\;x>0.5\;. \end{array}$$ (56)

**Fig 3:**
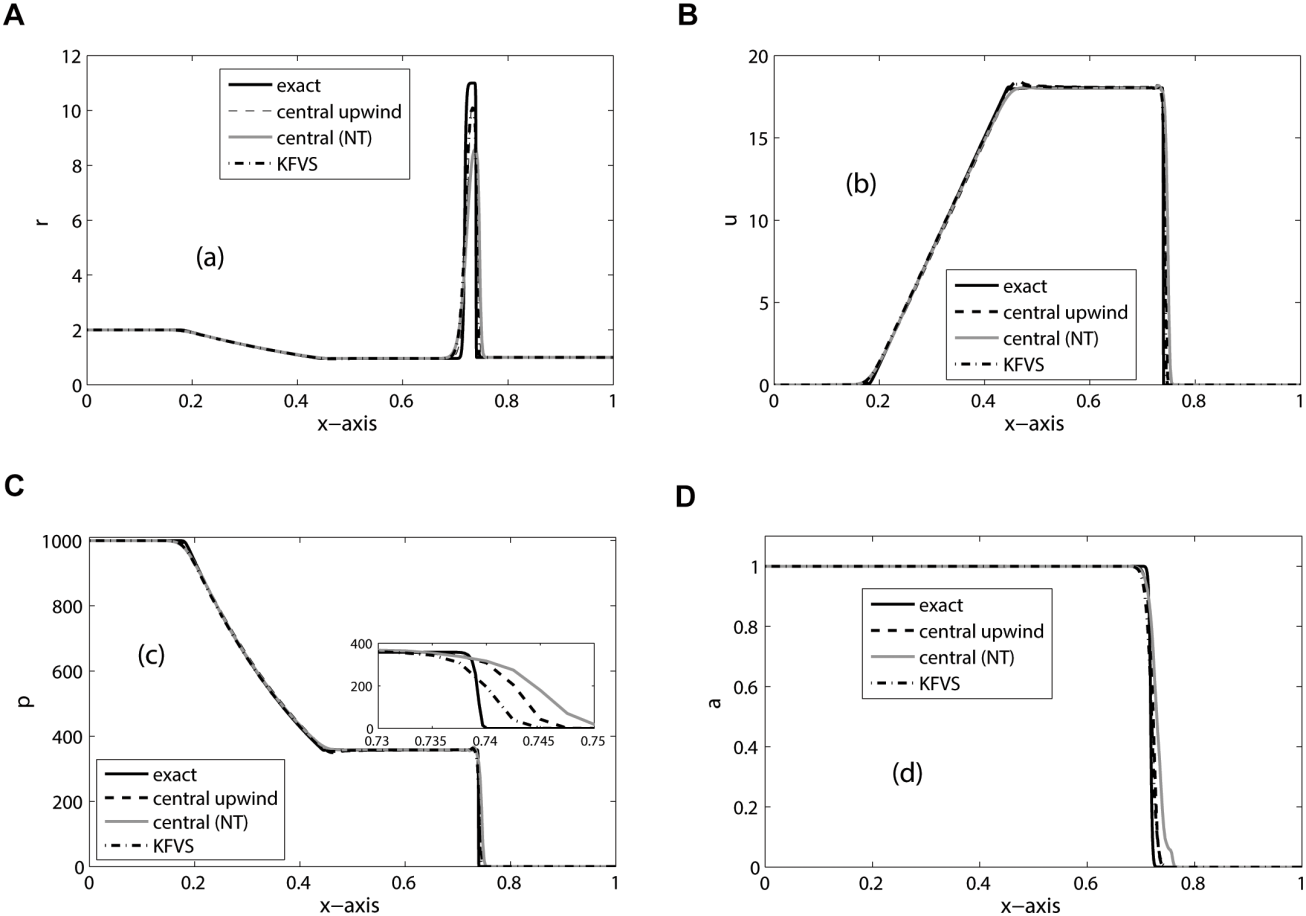
Results of Two-fluid mixture problem on 400 mesh cells at *t* = 0.012.

Here, *γ*_*L*_ = 1.4 and *γ*_*R*_ = 1.2, Π_*L*_ = 0 = Π_*R*_, and CFL = 0.5. This problem is also considered in [14]. It is a hard test problem for a numerical scheme. From the solution we can see a left moving rarefaction wave, a contact discontinuity, and a right moving shock wave. The right moving shock hits the interface at *x* = 0.5. The shock continues to move towards right and a rarefaction wave is created which is moving towards left. The results are simulated on 400 mesh cells and the final simulation time is taken as *t* = 0.012. The solutions are presented in the Fig 3. All the schemes give comparable results. However, from the zoomed graph it can be noted that KFVS scheme gives better resolution of peaks and discontinuities.

#### No-reflection problem

The initial data are given as

$$\begin{array}{c} (\rho ,u,p,\alpha )=(3.1748,9.435,100,1)\;,\;\text{if}\;\;\;x\leq 0.5, \end{array}$$ (57)

$$\begin{array}{c} (\rho ,u,p,\alpha )=(1,0,1,0)\;,\;\text{if}\;\;\;x>0.5\;. \end{array}$$ (58)

**Fig 4:**
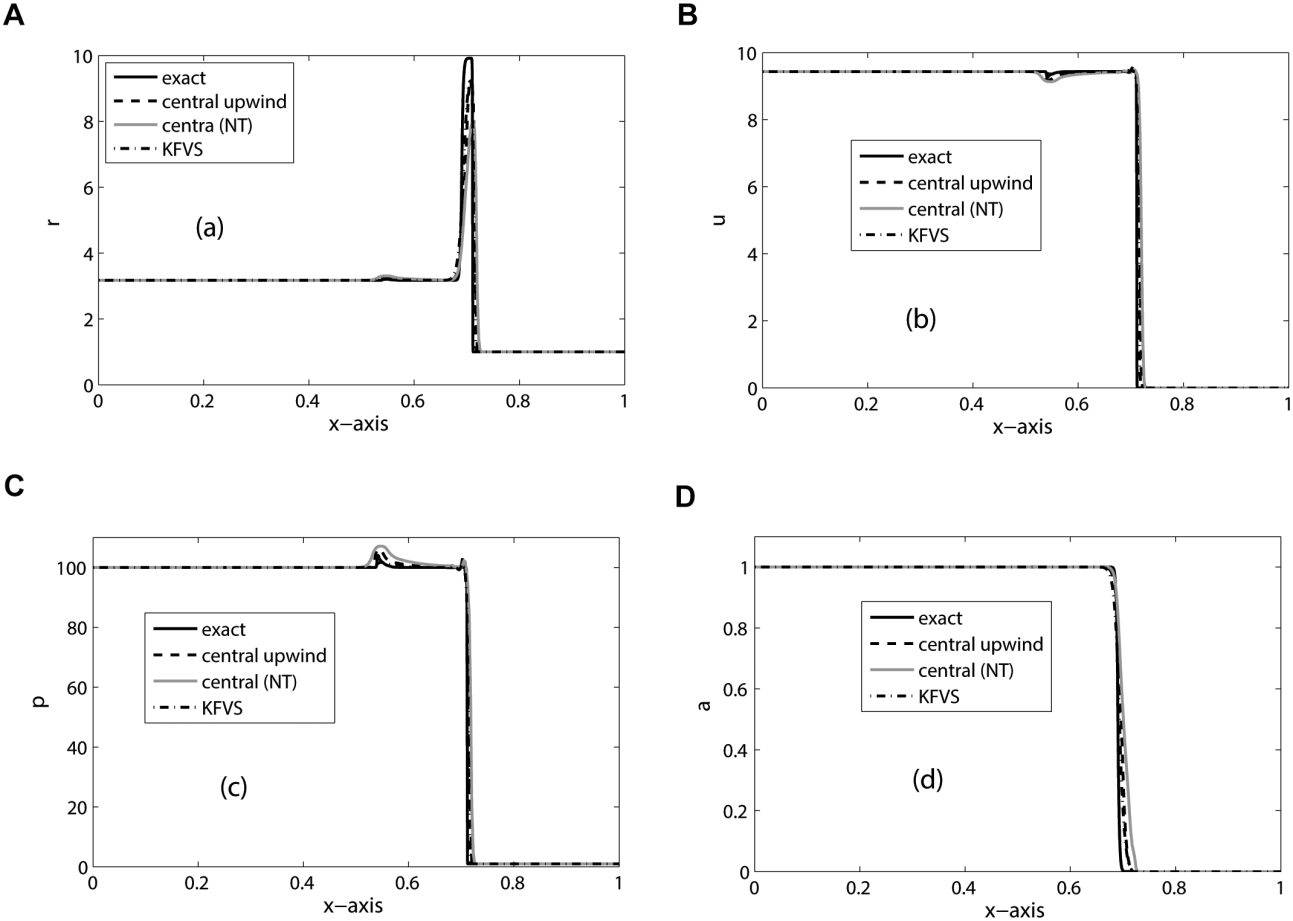
Results of No-reflection problem on 500 mesh cells at *t* = 0.02.

The ratio of specific heats are *γ*_*L*_ = 1.667 and *γ*_*R*_ = 1.2. Moreover, Π_*L*_ = 0 = Π_*R*_ and CFL = 0.4. We discretize the computational domain [0, 1] into 500 mesh cells and the final simulation time is *t* = 0.02. This is a hard test problem for a numerical scheme due to large jumps in pressure at the interface. The choice of pressure and velocity jump over the shock prevents the creation of a reflection wave. Therefore, a shock wave moves to the right. The results are depicted in Fig 4. Wiggles can be seen in the velocity and pressure plots of all schemes, representing small waves that are reflected to the left. However, unlike real velocity and pressure oscillations, these wiggles reduces on refined meshes. Similar type of wiggles are also reported in the results of [5].

#### Water-air mixture problem

This one-dimensional problem corresponds to the water-air mixture [5, 20]. The initial data are given as

$$\begin{array}{c} \left(\rho ,u,p,\alpha \right)=\left(525,0,10^{9},0.5\right)\;,\;\text{if}\;\;\;x\leq 0.5, \end{array}$$ (59)

$$\begin{array}{c} \left(\rho ,u,p,\alpha \right)=\left(525,0,10^{5},0.5\right)\;,\;\text{if}\;\;\;x>0.5\;. \end{array}$$ (60)

**Fig 5:**
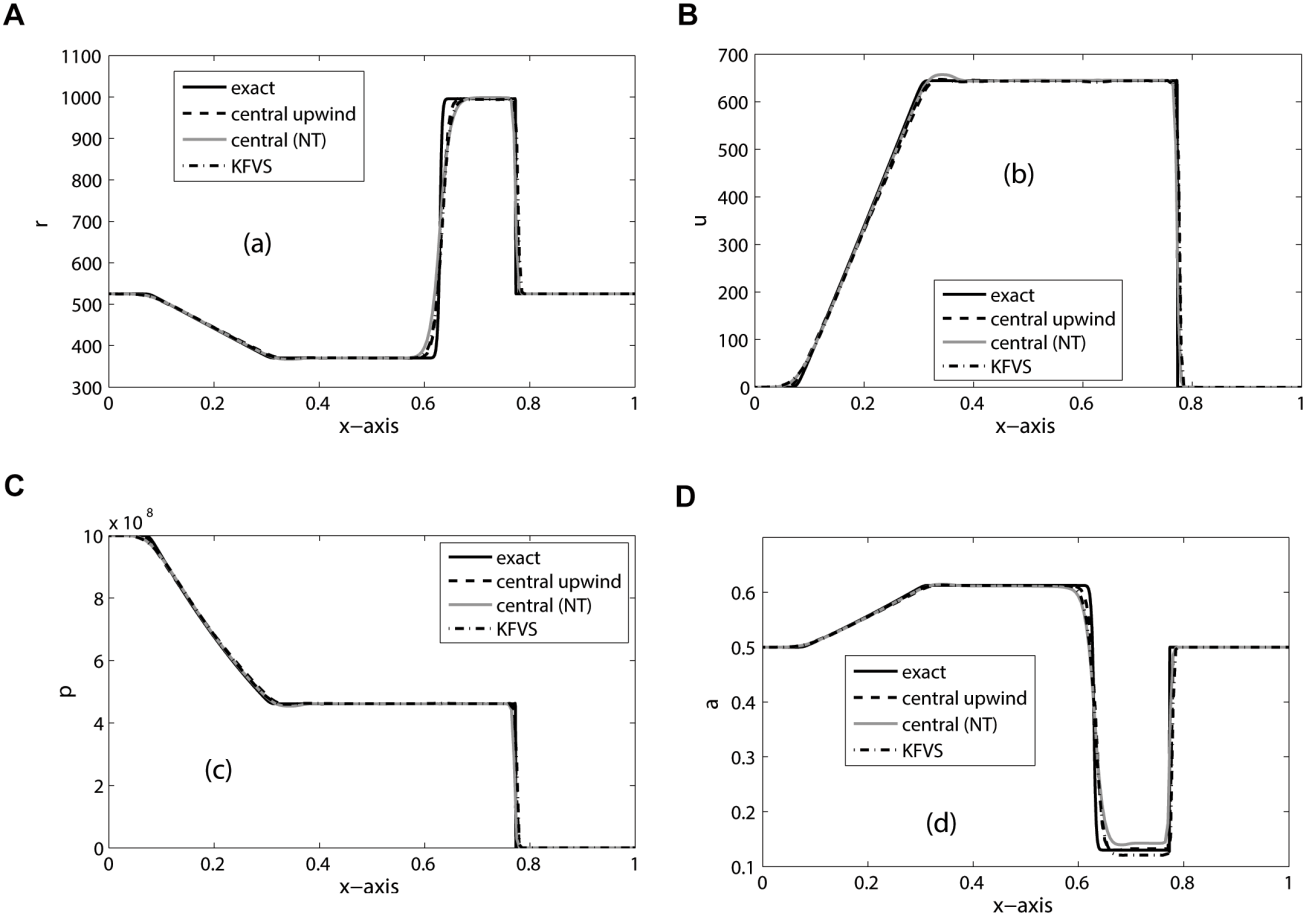
Results of Water-air mixture problem on 200 mesh cells at *t* = 200.

Here, *γ*_*L*_ = 1.4, *γ*_*R*_ = 4.4, Π_*L*_ = 0, Π_*R*_ = 6 × 10^8^ and CFL = 0.5. The domain [0, 1] is divided into 200 mesh cells and the final simulation time is *t* = 200. The numerical results are shown in Fig 5. Although the initial composition of the mixture is constant, it evolves in space and time. It can be observed that the three schemes give comparable results. Moreover, our results are in good agreement with the results in [20].

#### Water-air mixture problem

Again a one-dimensional water-air mixture problem [5, 20] is considered. However, this problem differs from the previous problem by allowing changes in the mixture composition. The initial data are given as

$$\begin{array}{c} \left(\rho ,u,p,\alpha \right)=\left(1,0,10^{9},0.2\right)\;,\;\text{if}\;\;\;x\leq 0.7, \end{array}$$ (61)

$$\begin{array}{c} \left(\rho ,u,p,\alpha \right)=\left(10^{3},0,10^{5},0.8\right)\;,\;\text{if}\;\;\;x>0.7\;. \end{array}$$ (62)

**Fig 6:**
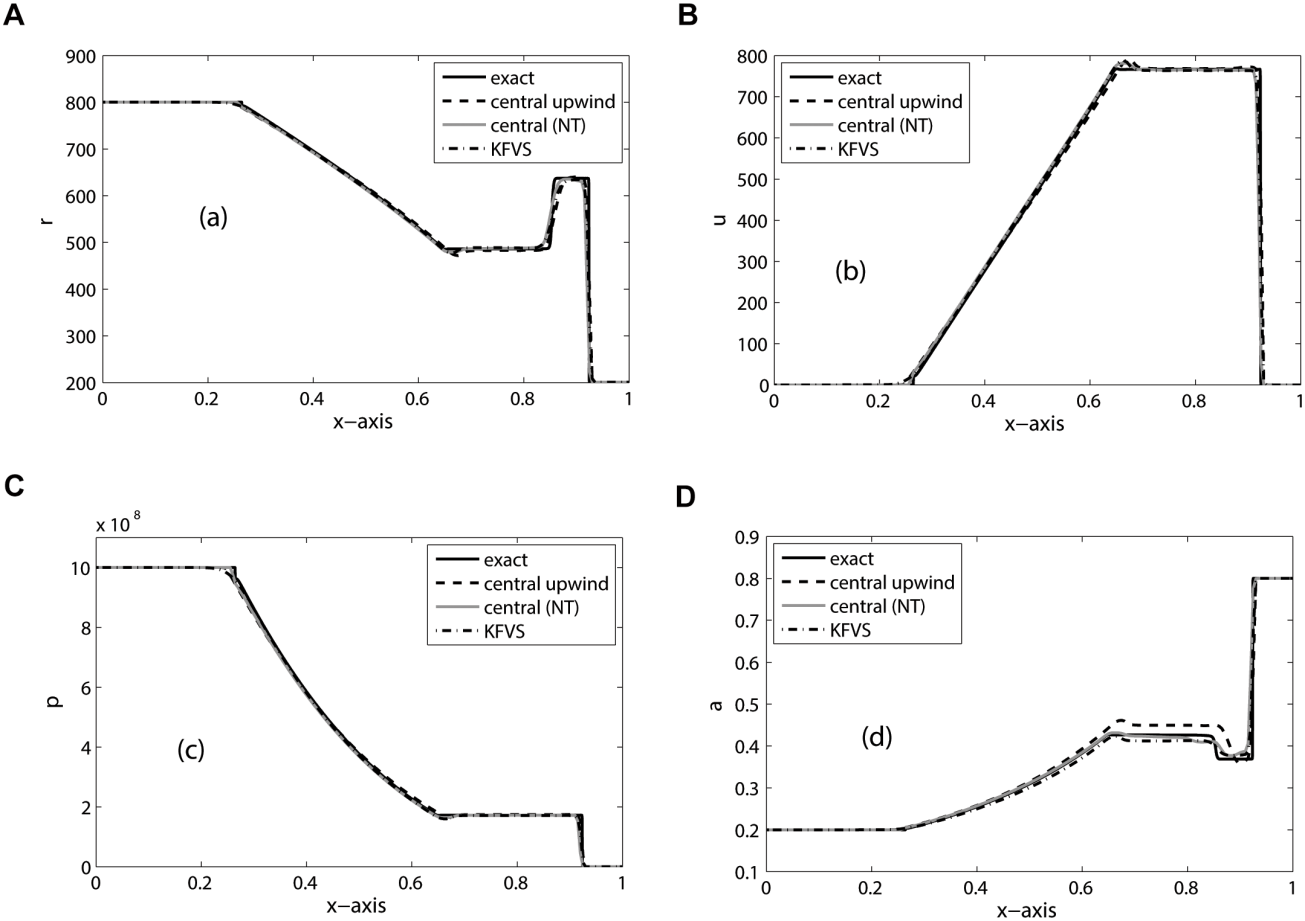
Results of Water-air mixture problem on 200 mesh cells at *t* = 200.

Here, *γ*_*L*_ = 1.4, *γ*_*R*_ = 4.4, Π_*L*_ = 0, Π_*R*_ = 6 × 10^8^ and CFL = 0.5. The results are simulated on 200 mesh cells and the final simulation time is *t* = 200. The numerical results are shown in Fig 6. From figure it can be noted that central upwind scheme give comparable results with other schemes. Moreover, the numerical results are in good agreement with those published in [20].

#### Translating two-fluid interface

The initial data for the problem are given as

$$\begin{array}{c} (\rho ,u,p,\alpha )=(1000,1,1,1)\;,\;\text{if}\;\;\;x\leq 0.25, \end{array}$$ (63)

$$\begin{array}{c} (\rho ,u,p,\alpha )=(1,1,1,0)\;,\;\text{if}\;\;\;x>0.25\;. \end{array}$$ (64)

**Fig 7:**
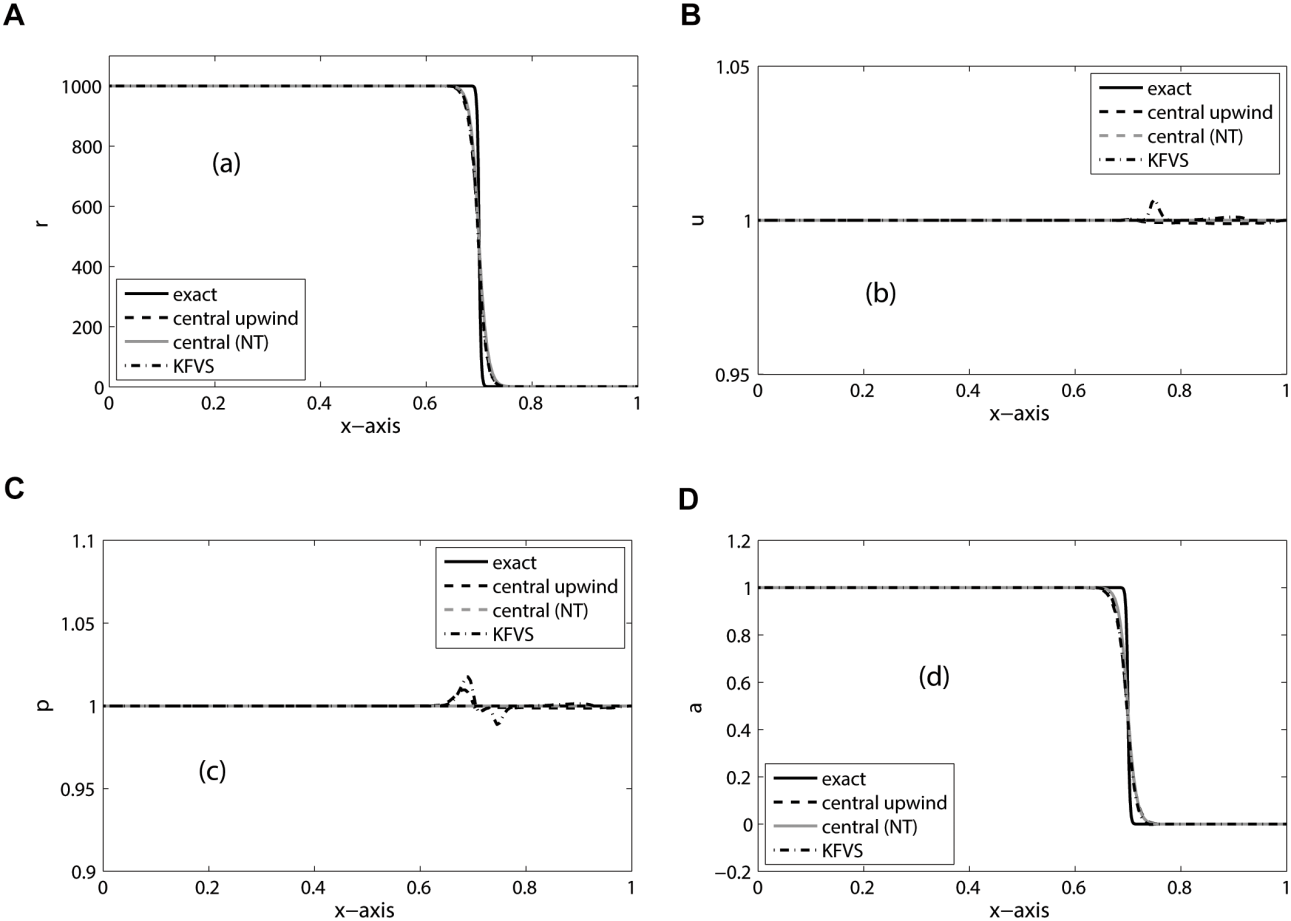
Results of Translating two-fluid interface problem on 200 mesh cells at *t* = 0.1.

The ratio of specific heats are given as *γ*_*L*_ = 1.4 and *γ*_*R*_ = 1.6. We have chosen 200 mesh cells and the final simulation time is *t* = 0.1. This problem is a contact discontinuity of water-air density ratio. The numerical results are shown in Fig 7. The same problem was also considered in [5, 6]. In this problem, both pressure and velocity are the same. Therefore, the interface is moving to the right with uniform speed and pressure. The numerical results show that KFVS scheme resolves the two-fluid interface very well as compared to the central scheme. Moreover, both velocity and pressure profiles are oscillation free.

### Two-dimensional test problems

**Fig 8:**
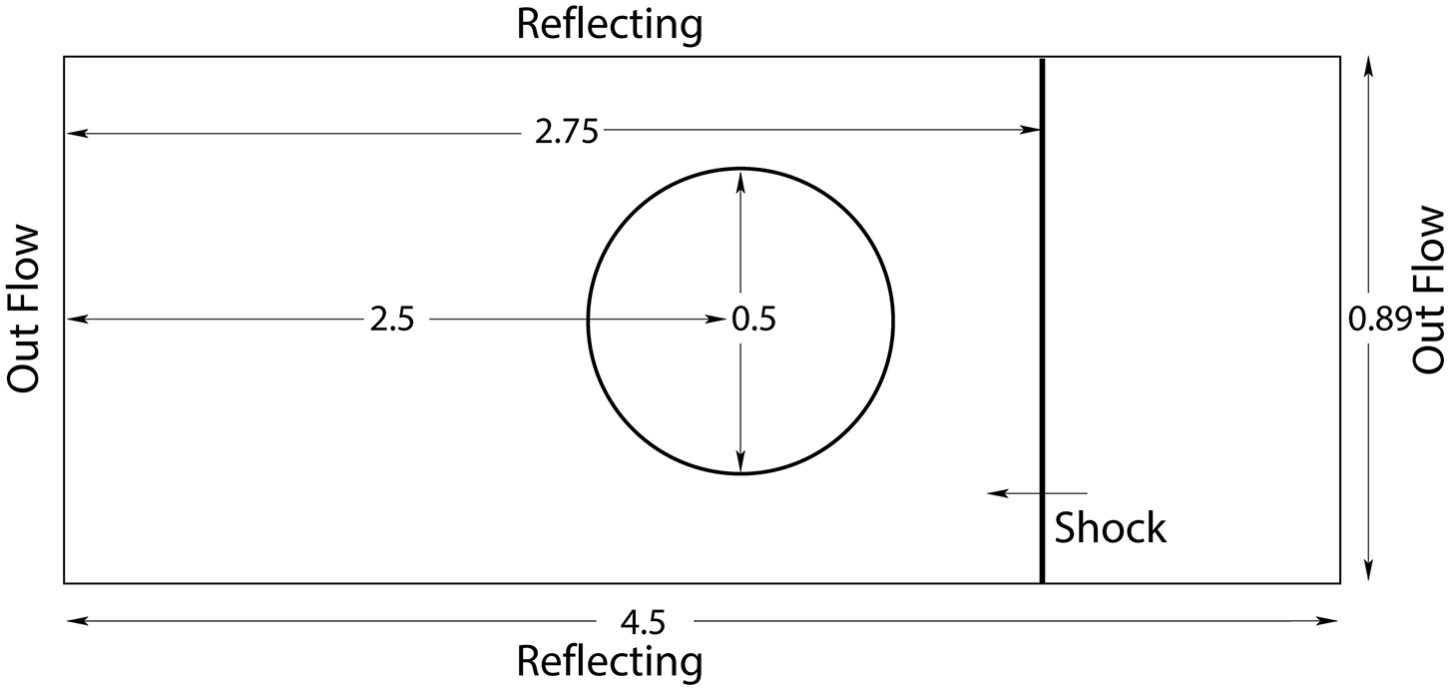
Sketch of computational domain.

To check the performance of proposed numerical scheme in two-dimensional space, we considered two test problems. In these problems the impact of a shock in air on a bubble of a lighter and a heavier gas is studied. Initially, Haas and Sturtevant [21] investigated these problems. Later, Quirk and Karni [22], Marquina and Mulet [23], Kreeft and Koren [5] and Wackers and Koren [6] also discussed these test cases. A schematic computational setup for these two problems is sketched in Fig 8. A shock tube of length 4.5 and width 0.89 is considered. The top and bottom walls of the tube are solid reflecting walls, while both ends of the tube are open. A cylinder of very thin cellular walls filled with gas is placed inside the tube. A shock wave is generated in the right end of the shock tube and is moving from right to left. After hitting by shock, the walls of the cylinder ruptures and the shock interacts with the gas of the cylinder. Due to fast interaction both gases do not mix in large amount, leading to a two-fluid flow problem. As the shock approaches to the surface of the bubble a reflected shock is generated from the surface of the bubble which moves towards right back in the air. At later time, this interaction become more and more complicated. The shock continues to move towards right in the air after passing through bubble and produces secondary reflected waves in the bubble when it hits the surface of the bubble.

The wave patterns generated by interaction are strongly depending on the density of the gas inside the bubble. However, some of the waves can be observed in almost all cases [5, 6]. Here, a light helium gas and a heavy R22 gas are considered inside the cylindrical bubble.

#### Helium bubble

In this problem, we study the interaction of *Ms* = 1.22 planar shock, moving in air, with a cylindrical helium bubble contaminated with 28% of air. The bubble is assumed to be in thermodynamical and mechanical equilibrium with the surrounding air. The initial data are given as

$$\begin{array}{ccc} \left(\rho ,u,v,p,\gamma \right) & = & (1.40000,0.0,0.0,1.0,1.4)\;,\;\text{pre-shock}\;\text{air},\\ \left(\rho ,u,v,p,\gamma \right) & = & (1.92691,-0.33361,0.0,1.5698,1.4)\;,\;\text{post-shock}\;\text{air},\\ \left(\rho ,u,v,p,\gamma \right) & = & (0.25463,0.0,0.0,1.0,1.648)\;,\;\text{helium}. \end{array}$$

**Fig 9:**
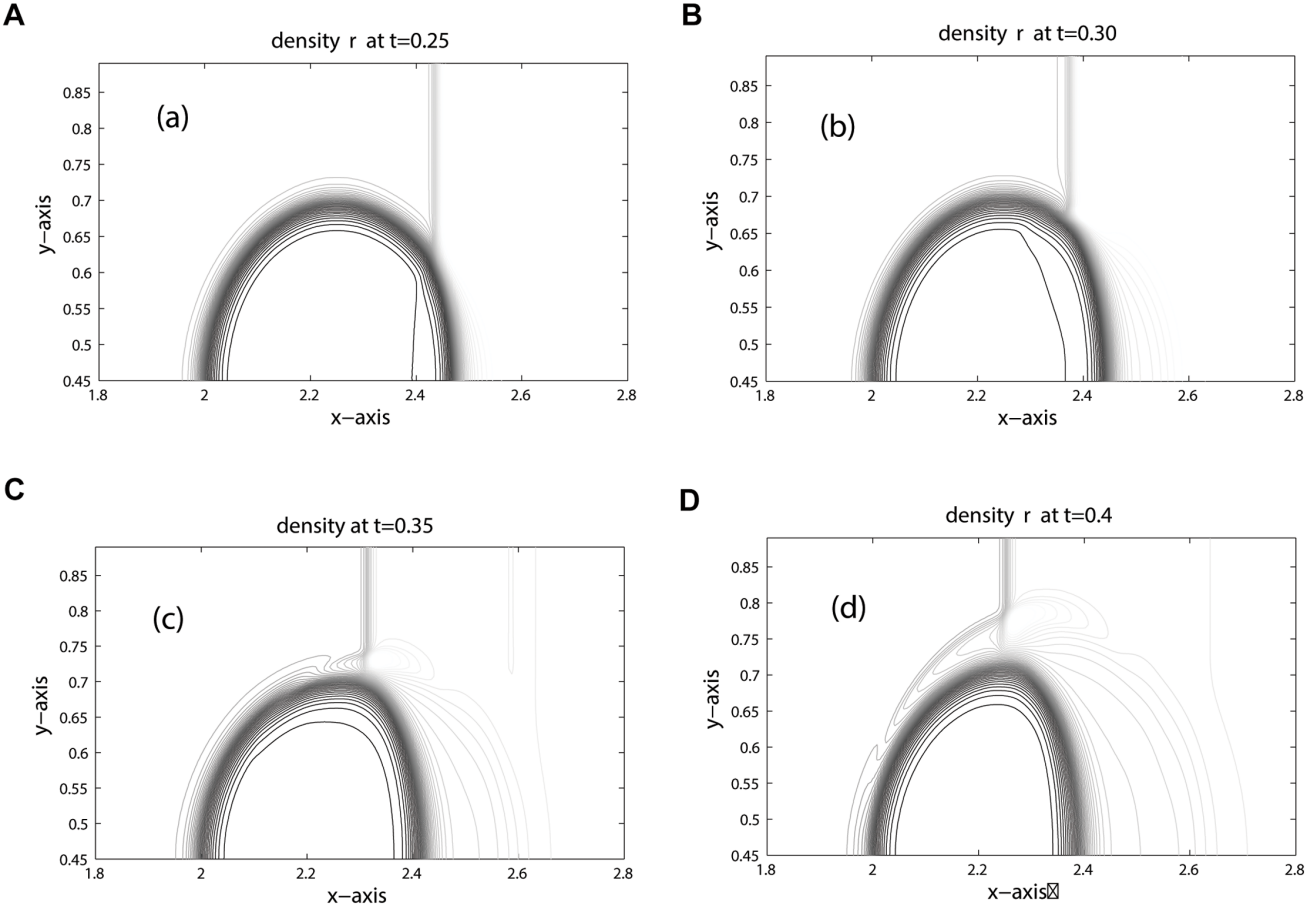
Density contours of Helium Bubble problem (shock hitting helium bubble).

**Fig 10:**
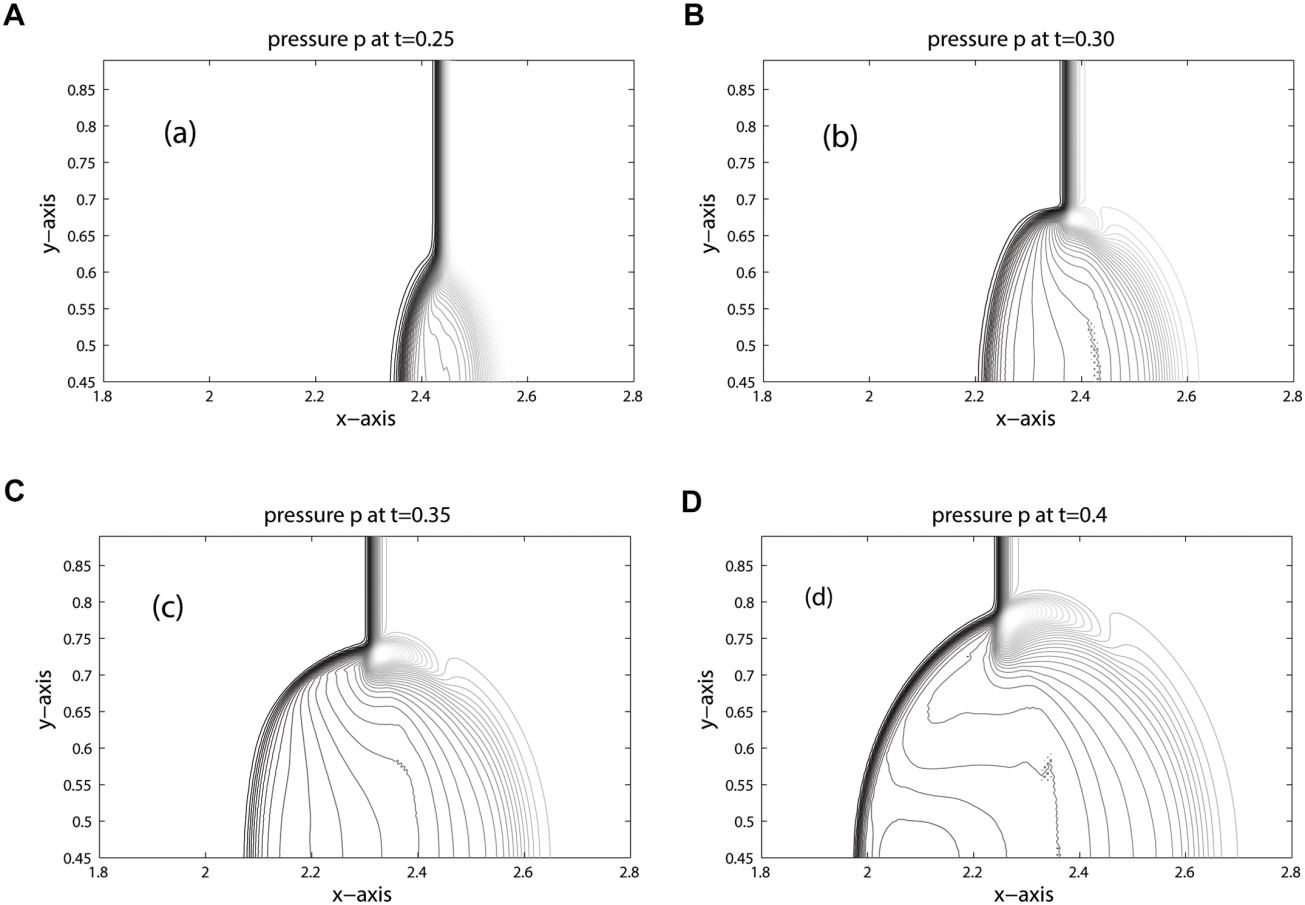
Pressure contours of Helium Bubble problem (shock hitting helium bubble).

**Fig 11:**
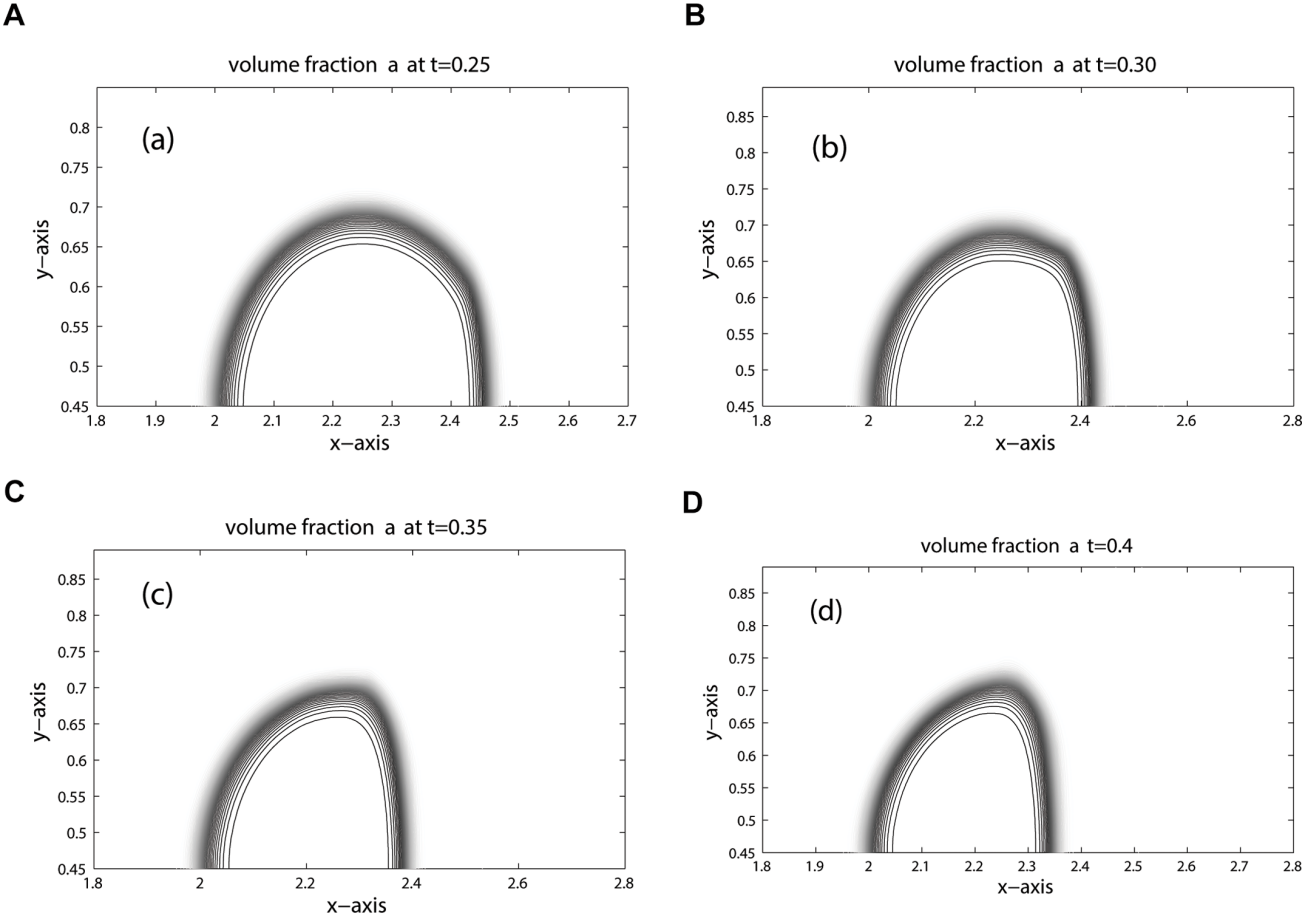
Volume fraction contours of Helium Bubble problem (shock hitting helium bubble).

**Fig 12:**
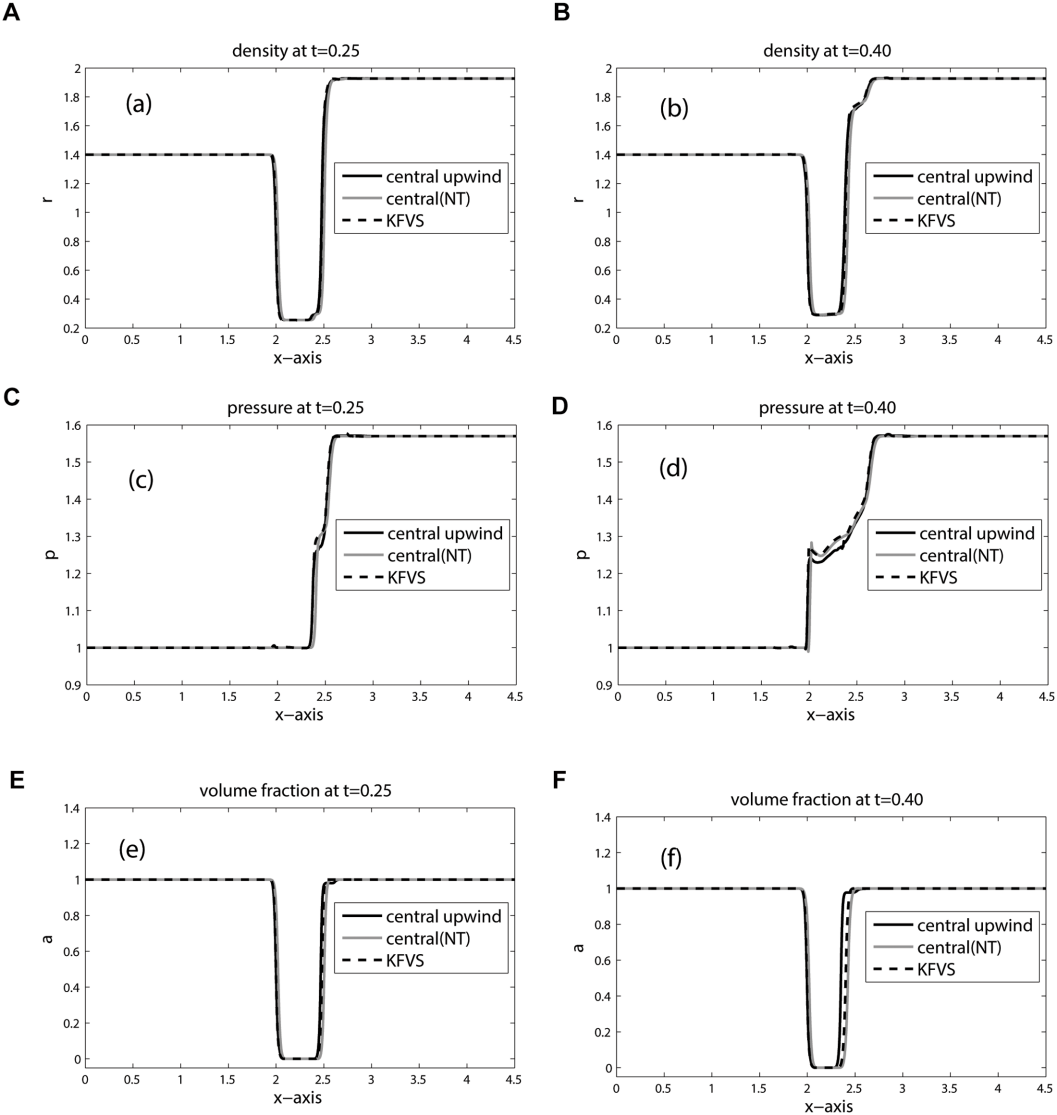
Plots along *y* = 0.445 of Helium Bubble problem (shock hitting helium bubble).

The position of key features occurred during the time evolution are well explained in [5, 6, 23]. Therefore, we omit discussion on these features. The computational domain is discretized into 800 × 200 mesh cells. The contours for density, pressure and volume fraction are depicted in Figs 9, 10 and 11 at time: 0.25, 0.30, 0.35, 0.40. These results agree closely with the plots given in [5, 6, 21, 22] at times: 32 *μ*s, 52 *μ*s, 62 *μ*s, 82 *μ*s. In Figs 10 and 11 the contours of pressure and volume fraction show a perfect splitting of the pressure waves and the interface. The shocks and interface are sharp during the simulation. As observed in [6], the last interface is slowly bending inwards in Fig 11. The phenomena will continue at later times until the bubble split in two vortices. The comparison between KFVS and central schemes can be clearly observed in Fig 12.

#### R22 bubble

Here, the same *Ms* = 1.22 planar shock moving in air hits a cylindrical R22 bubble which has higher density and lower ratio of specific heats than air. This results in about two times lower speed of sound. For more details, the reader is referred to [5, 6]. The initial data are given as

$$\begin{array}{ccc} \left(\rho ,u,v,p,\gamma \right) & = & (1.40000,0.0,0.0,1.0,1.4)\;,\;\text{pre-shock}\;\text{air},\\ \left(\rho ,u,v,p,\gamma \right) & = & (1.92691,-0.33361,0.0,1.5698,1.4)\;,\;\text{post-shock}\;\text{air},\\ \left(\rho ,u,v,p,\gamma \right) & = & (4.41540,0.0,0.0,1.0,1.249)\;,\;\text{R22}. \end{array}$$

**Fig 13:**
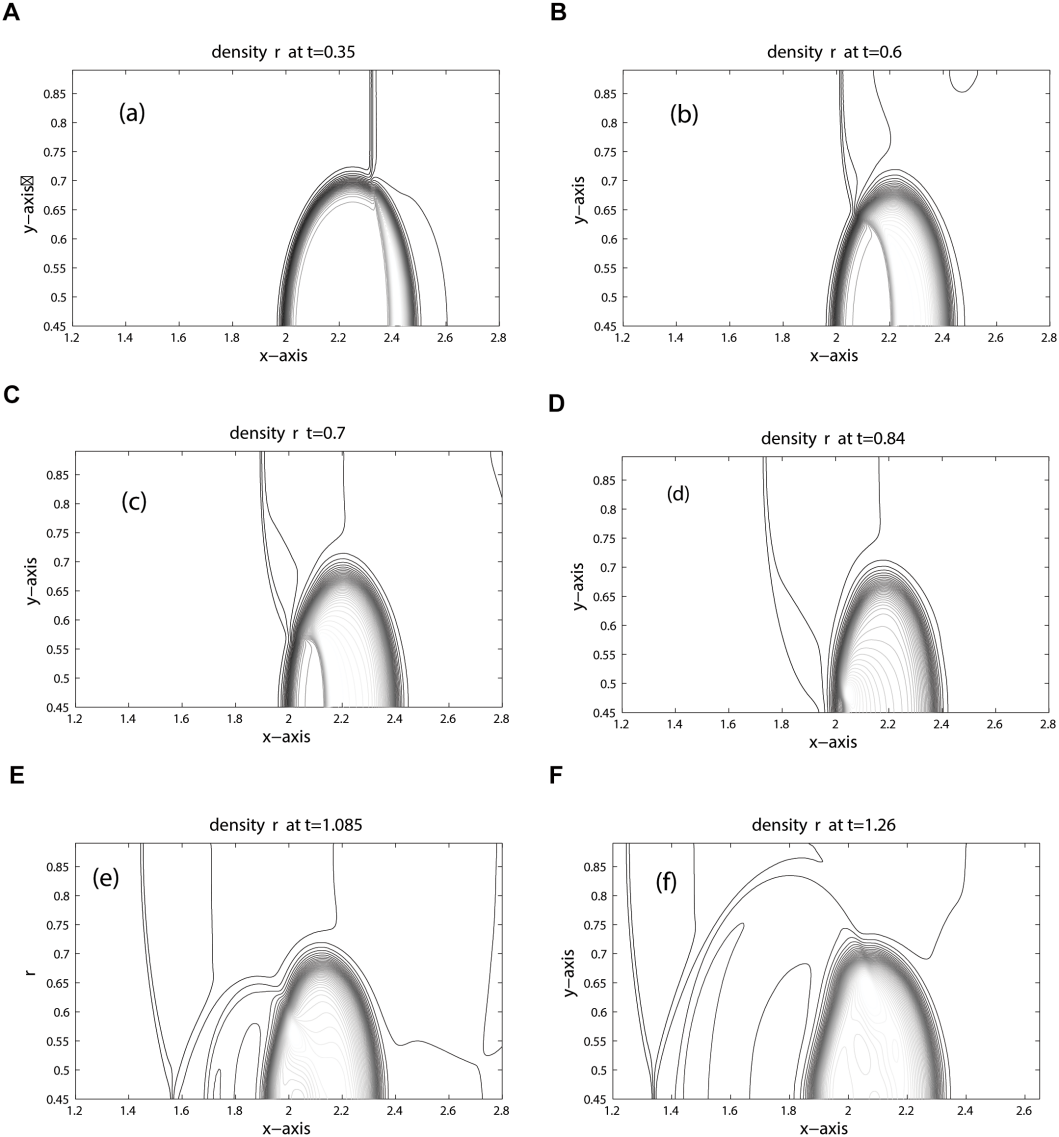
Density contours of R22 Bubble problem (shock hitting R22 bubble).

**Fig 14:**
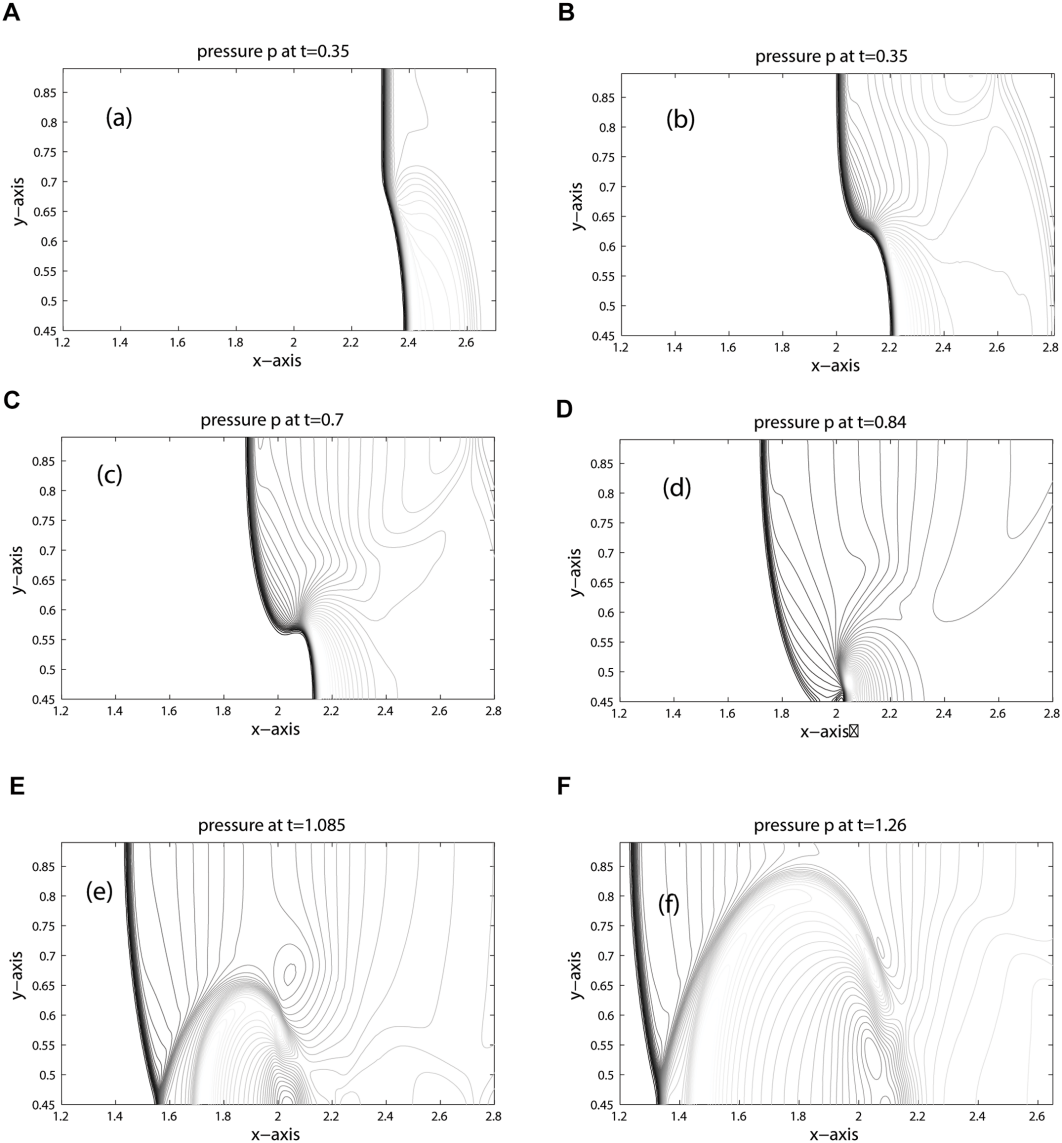
Pressure contours of R22 Bubble problem (shock hitting R22 bubble).

**Fig 15:**
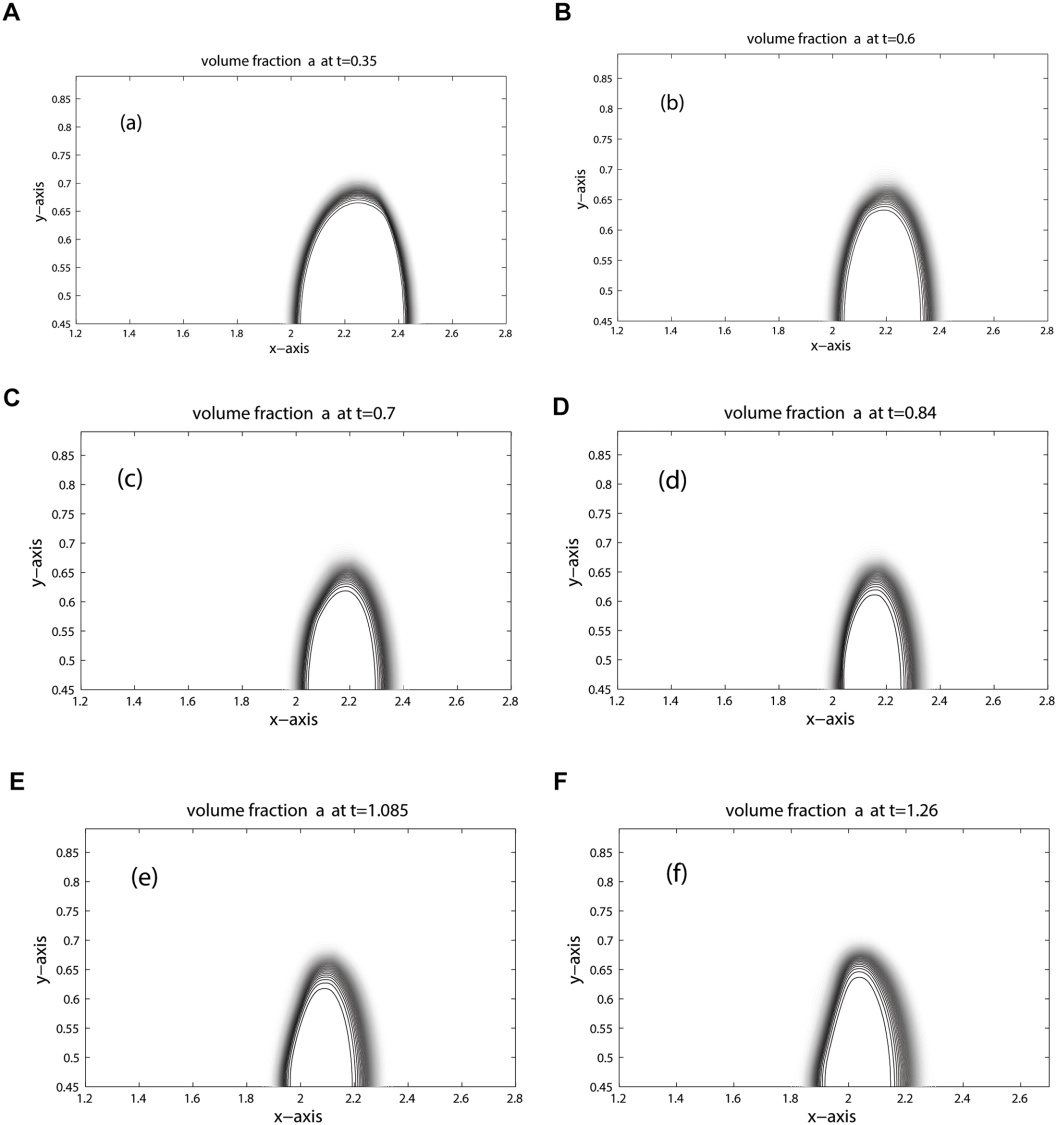
Volume fraction contours of R22 Bubble problem (shock hitting R22 bubble).

**Fig 16:**
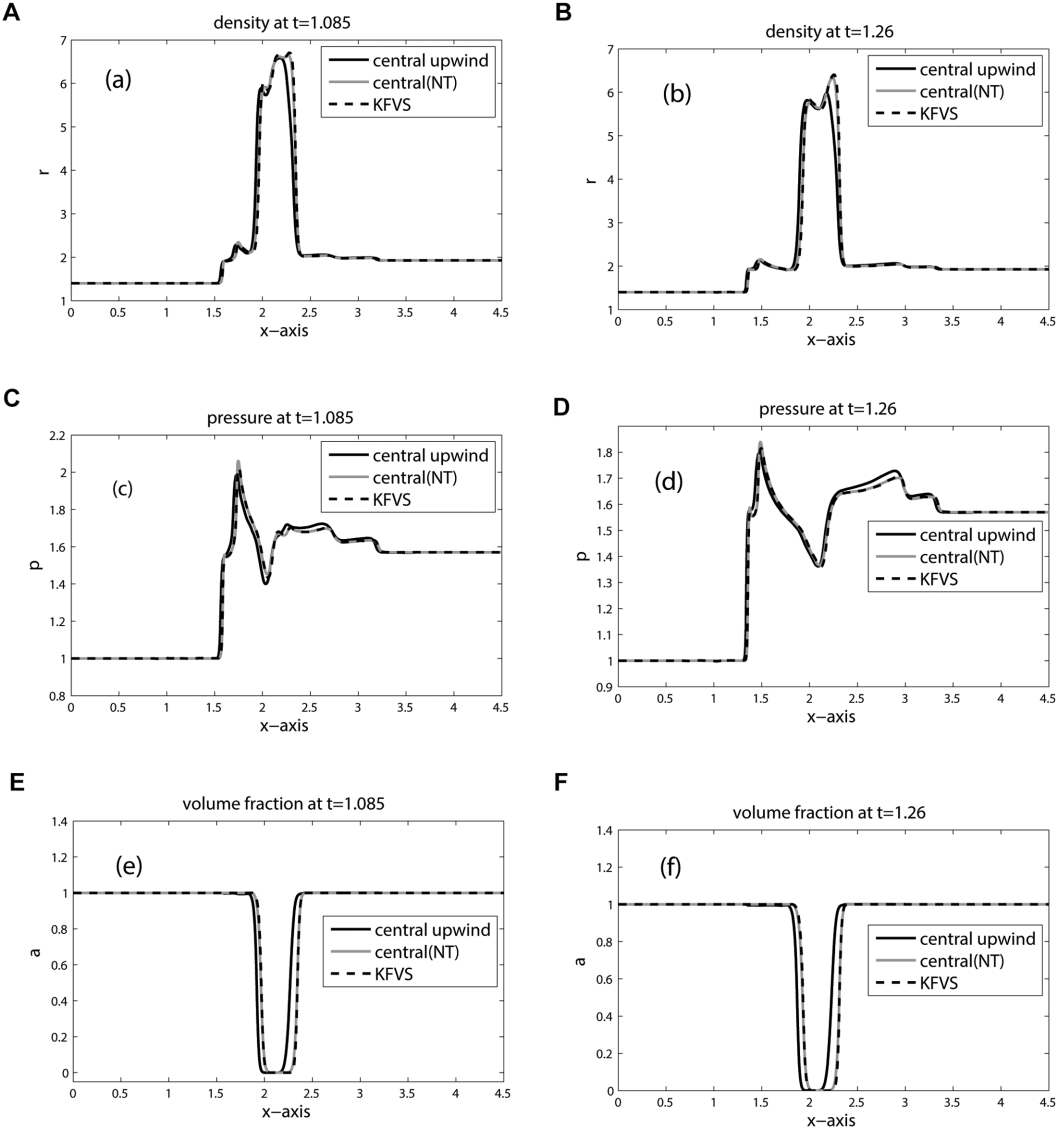
Plots along *y* = 0.445 for R22 Bubble problem (shock hitting R22 bubble).

The computational domain is discretized into 800 × 200 mesh cells. Due to the lower speed of sound, the shock in the bubble and the refracted shock lag behind the incoming shock. Moreover, due to the circular shape of the bubble the refracted, reflected and shock waves are curved. The results for density, pressure and volume fraction are displayed in Figs 13, 14 and 15 at times: 0.35, 0.60, 0.70, 0.84, 1.085, 1.26. These results shows good agreement with the results [5, 6, 21, 22] at times: 55 *μ*s, 115 *μ*s, 135 *μ*s, 187 *μ*s, 247 *μ*s, 318 *μ*s. The splitting in pressure and interface is observed in flow pattern of density contours. Moreover, no wiggles are visible in our results and the pressure is continuous over the interface. Hence, the numerical results of our scheme reflect all key features as explained in [5, 6, 21]. Fig 16 compare the results of KFVS and central schemes along the centerline *y* = 0.445. Both the schemes have comparable accuracy.

## Conclusions

A central upwind finite volume scheme was extended to solve the compressible two-phase reduced five-equation model in one and two-dimensional space. The suggested scheme is based on the estimation of cell averages by using the information of local propagation speed. In two-dimensional space the scheme is implemented in a usual dimensionally split manner. The non-differential part of the source terms are approximated by the cell averaged values, whereas the differential part terms are approximated similar to the convective fluxes. To preserve the positivity of the scheme a min-mod non-linear limiter is used. To achieve the second order accuracy in time a TVD Runge-Kutta method is utilized. For validation, the results of the proposed numerical scheme are compared qualitatively and quantitatively with those of KFVS and staggered central schemes. Good agreements were observed in the results of all three schemes. It was found that in some test problems central upwind scheme produced more accurate results, while KFVS scheme performed well in other problems. Perhaps, this is due to the reason that both the schemes are upwind biased. The staggered central scheme was found diffusive in all test problems.
